# Outcomes following severe hand foot and mouth disease: A systematic review and meta-analysis

**DOI:** 10.1016/j.ejpn.2018.04.007

**Published:** 2018-09

**Authors:** Eben Jones, Timesh D. Pillay, Fengfeng Liu, Li Luo, Juan Carlos Bazo-Alvarez, Chen Yuan, Shanlu Zhao, Qi Chen, Yu Li, Qiaohong Liao, Hongjie Yu, H. Rogier van Doorn, Saraswathy Sabanathan

**Affiliations:** a University Hospital Lewisham, National Health Service, London, UK; b Division of Infectious Disease, Key Laboratory of Surveillance and Early Warning on Infectious Disease, Chinese Center for Disease Control and Prevention, Beijing, China; c Methodology Research Group, Department of Primary Care and Population Health, University College London (UCL), London, UK; d Zhoushan Center for Disease Control and Prevention, Zhoushan, Zhejiang, China; e Hunan Provincial Center for Disease Control and Prevention, Changsha, Hunan, China; f Hubei Provincial Center for Disease Control and Prevention, Changsha, Hunan, China; g School of Public Health, Fudan University, Key Laboratory of Public Health Safety, Ministry of Education, Shanghai, China; h Oxford University Clinical Research Unit, Ha Noi, Viet Nam; i Nuffield Department of Medicine, Centre for Tropical Medicine and Global Health, Oxford University, Oxford, UK

**Keywords:** Hand, foot and mouth disease, Enterovirus A71, Neurological, Encephalitis, Sequelae, Outcome, Meta-analysis, Systematic review

## Abstract

**Background:**

Hand, foot and mouth disease (HFMD) caused by enterovirus A71 (EV-A71) is associated with acute neurological disease in children.

This study aimed to estimate the burden of long-term sequelae and death following severe HFMD.

**Methods:**

This systematic review and meta-analysis pooled all reports from English and Chinese databases including MEDLINE and Wangfang on outbreaks of clinically diagnosed HFMD and/or laboratory-confirmed EV-A71 with at least 7 days' follow-up published between 1st January 1966 and 19th October 2015.

Two independent reviewers assessed the literature.

We used a random effects meta-analysis to estimate cumulative incidence of neurological sequelae or death.

Studies were assessed for methodological and reporting quality.

PROSPERO registration number: 10.15124/CRD42015021981.

**Findings:**

43 studies were included in the review, and 599 children from 9 studies were included in the primary analysis.

Estimated cumulative incidence of death or neurological sequelae at maximum follow up was 19.8% (95% CI:10.2%, 31.3%).

Heterogeneity (Iˆ2) was 88.57%, partly accounted for by year of data collection and reporting quality of studies.

Incidence by acute disease severity was 0.00% (0.00, 0.00) for grade IIa; 17.0% (7.9, 28.2) for grade IIb/III; 81.6% (65.1, 94.5) for grade IV (p = 0.00) disease.

**Conclusions:**

HFMD with neurological involvement is associated with a substantial burden of long-term neurological sequelae. Grade of acute disease severity was a strong predictor of outcome.

Strengths of this study include its bilingual approach and clinical applicability.

Future prospective and interventional studies must use rigorous methodology to assess long-term outcomes in survivors.

**Funding:**

There was no specific funding for this study. See below for researcher funding.

## Highlights

• Severe hand, foot and mouth disease (HFMD) is associated with CNS involvement.
• Severe HFMD is associated with a significant burden of neurological sequelae.
• Those with more severe acute disease are at higher risk of developing sequelae.
• Future prospective and interventional studies should assess long-term outcomes.

## 1 Introduction

Hand foot and mouth disease (HFMD) is a clinical entity consisting of fever and vesicular rash on the palmar and plantar aspects of the hands and feet with or without herpangina, ulcers on the buccal mucosa. HFMD outbreaks, seen in preschool children, are usually benign and self-limiting. However, since the late 1990s, outbreaks associated with neurological complications in Malaysia and Taiwan heralded a new paediatric encephalitis threat in the region.^1^^,^^2^ HFMD has been associated with enterovirus A infection, and more severe clinical outcomes are associated with enterovirus A71 (EV-A71) specifically, but recent outbreaks in China suggest other pathogens may also be associated with neurological complications.^3^, ^4^, ^5^ Ongoing outbreaks in China and Vietnam, and increasing reports of cases with severe manifestations in Europe, make HFMD a disease of regional and global importance.^6^, ^7^, ^8^, ^9^.

In China alone, between 2008 and 2012, 6.5 million children were diagnosed with HFMD and more than 2200 died. Annual incidence of both disease and death are increasing.^10^ A recent meta-analysis has estimated a 1.7% pooled case-mortality rate for clinically confirmed HFMD,^11^ substantially higher than polio in which approximately 4% of symptomatic cases result in acute flaccid paralysis (AFP) of whom 2–5% of children and 15–30% of adults die,^12^ validating its recent description as “the new polio”.^13^^,^^14^.

Aseptic meningitis, brainstem encephalitis, encephalomyelitis, cerebellar ataxia, AFP and life threatening cardiopulmonary failure (CPF) have been reported as HFMD-associated neurological complications, ^14^ best delineated in the World Health Organisation (WHO) grading system of acute severity [Graphic 1]. But in survivors of severe HFMD it is not clear what the burden and course of neurological, cognitive and developmental sequelae are, nor which specific areas are impaired.^15^^,^^16^ Furthermore, whilst a number of studies have identified risk factors for acute disease severity (younger age of onset, high and prolonged fever and neurological involvement),^14^,^17^ similar markers predictive of long-term morbidity are lacking. The adequate management of future outbreaks is contingent on understanding where to focus resources to prevent and treat acute disease as well as ameliorate long-term disease burden. The future morbidity and mortality of this emerging infection is even less clear. Recent outbreaks have been heterogeneous in aetiology, size, mortality rate and hospital burden.^5^^,^^18^[Graphic 2] [Graphic 3].

**Graphic 1:**
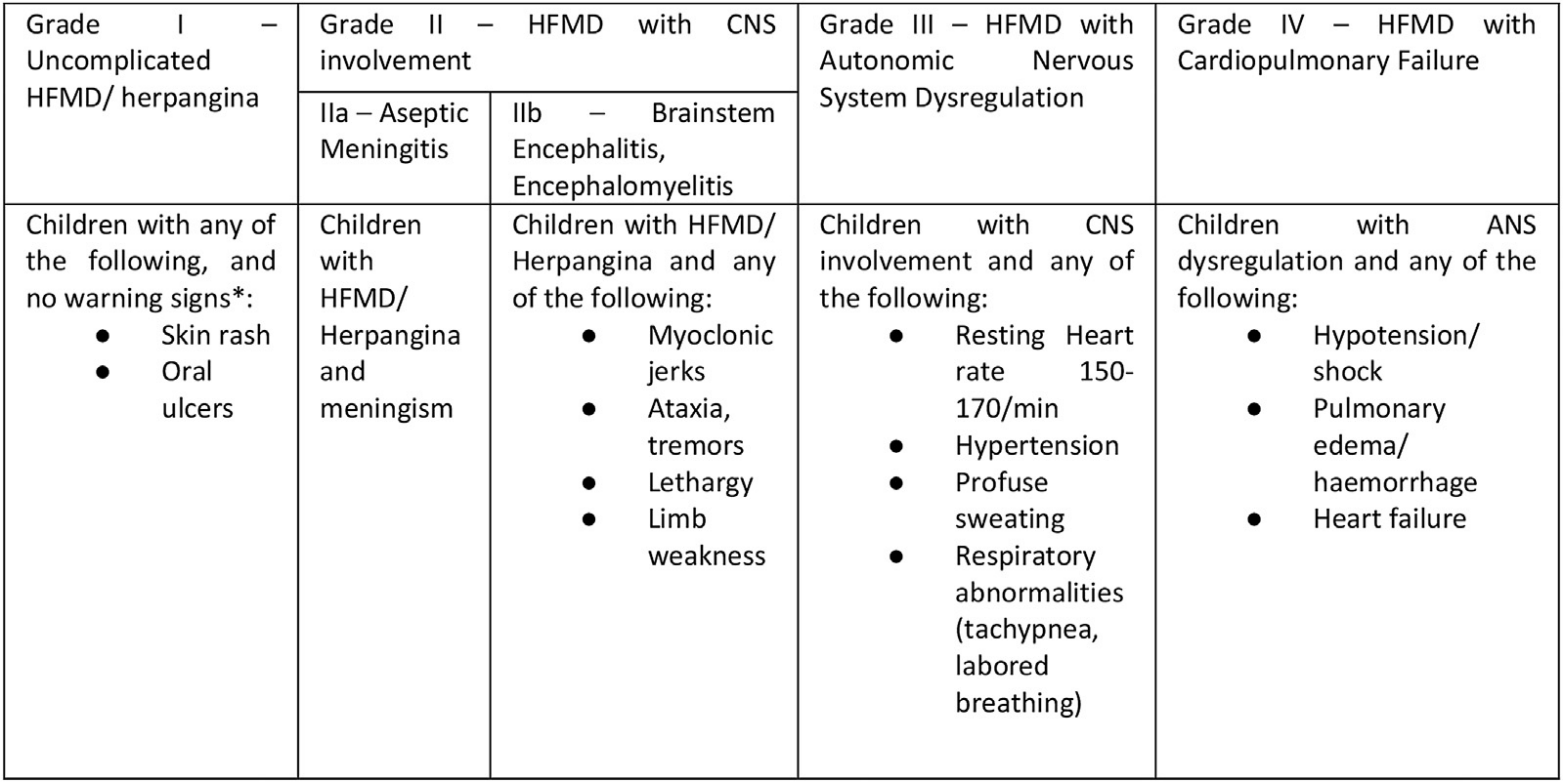
WHO disease severity classification by clinical criteria.

Adapted from ‘A guide to clinical management and public health response for Hand, Foot and Mouth Disease’.^19^

**Graphic 2:**
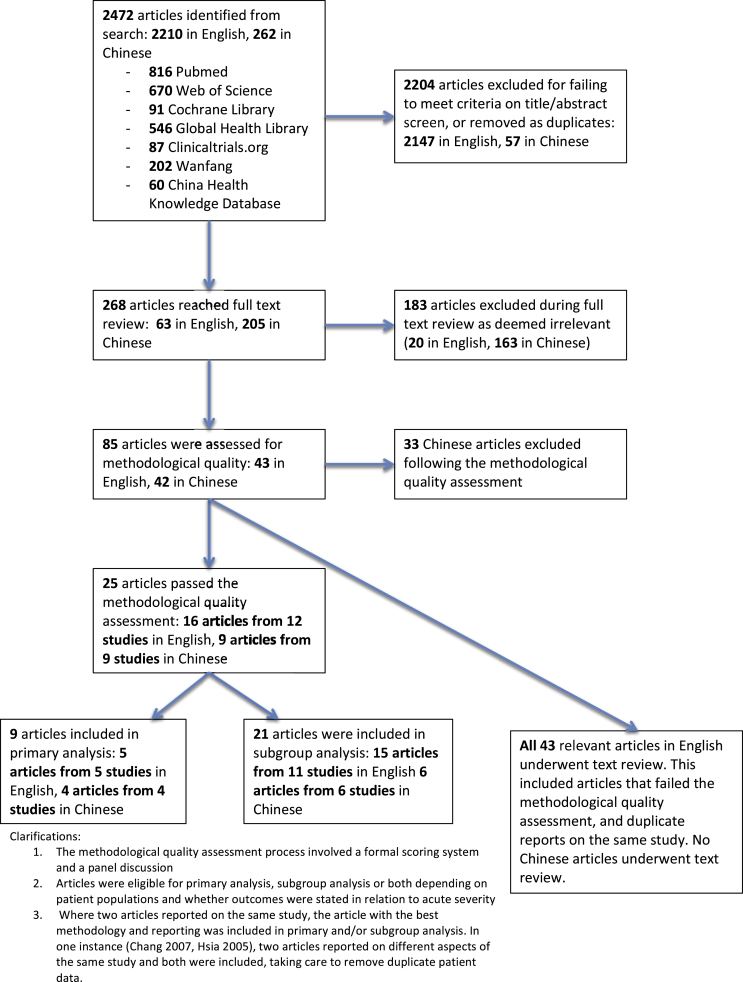
Flowchart of study exclusion and data extraction.

**Graphic 3:**
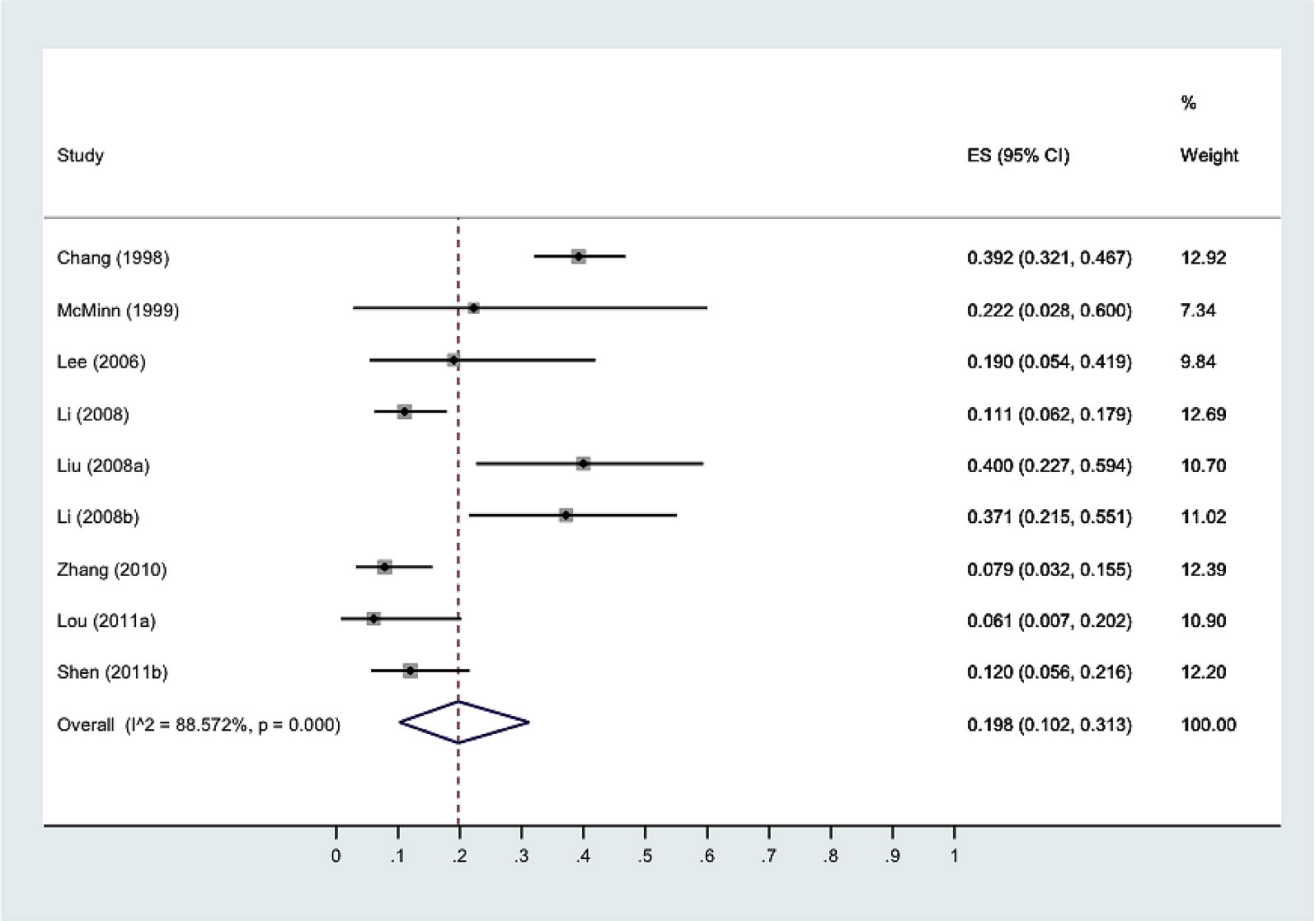
Forest plot with estimates for risk of death or neurological sequelae at maximum follow-up after severe HFMD. *Author (year of first data collection).

This systematic review and meta-analysis aims to describe the risk of long-term outcomes in cases of severe HFMD disease; the risk by World Health Organisation clinical severity grade,^13^^,^^19^ demographics, MRI findings and interventions; and qualitative lessons from the literature.

## 2 Methods

### 2.1 Search strategy and selection criteria

This systematic review and meta-analysis was performed and reported according to the PRISMA statement.^20^ We included studies that reported on all outcomes following severe HFMD, defined as WHO grade IIa-IV in paediatric populations, where cases were defined clinically (gold standard) and/or using standard laboratory techniques, with minimum seven days' follow-up and from an outbreak context. We included studies published in both English and Chinese. We worked in English-speaking (TP, EJ, RvD, SS) and Mandarin-speaking teams (FL, LL, CY, SZ, QC, YL, QL and HY) that collaborated closely throughout to ensure methodological consistency.

We compiled a Boolean search term [available in Appendix 1] in English using the validated hedge term for *children*^21^; descriptions of the exposure (*hand, foot and mouth disease*, *enterovirus A71* and all other permutations); of outcome as concept (e.g. *follow-up*, *outcome*); and of outcome type (e.g *neurodevelopment*, *weakness*) including relevant medical subject headings. It was translated and modified for use in Chinese databases, and validated by employing scoping searches and consulting with experts in the field.

In English, we searched MEDLINE, Embase, Web of Science (including conference proceedings), Cochrane Library and Global Health Library (limited to developing country regions). In Chinese, we searched Wangfang and China Hospital Knowledge Database, along with other databases to identify relevant clinical trial and grey literature [Appendix 1]. Randomised controlled trials (RCTs), observational studies, cohort studies, cross sectional studies, case series and reports and grey literature were included. We included studies published between 1st January 1966 and 19th October 2015.

### 2.2 Data extraction and quality assessment

Title and abstract screening was performed by two independent reviewers, with consensus decision in cases of disagreement. Screening of clinical trials and grey literature was performed by one team member. All studies passing title and abstract screening were subjected to full text review, independently performed by two reviewers. Any disagreements about final inclusion were resolved by a third reviewer. We scrutinised publications for duplicate data.

We used a score based on STROBE guidelines^22^ to assess risk of bias and quality of reporting [Appendix 2]. Publications were selected for primary analysis if they were representative of our population of interest, studied severe HFMD defined as WHO grade IIa to IV, reported on follow-up beyond 7 days after acute disease onset and demonstrated adequate methodology and reporting. Publications focusing on acute severity subgroups or describing outcomes divided as such were selected for subgroup analysis. Where loss to follow-up was reported, these children were excluded from the analysis. All English articles, including duplicates and those with a poor quality score, were eligible for text review. Where we established that data from the same study was reported in two or more publications, we employed a pragmatic approach to include data from the strongest, most relevant article.

### 2.3 Data synthesis and analysis

Data regarding study identification; number of participants and number with long-term outcome; demographics, acute disease severity as per WHO classification [Graphic 1], MRI findings and interventions; duration of follow-up; qualitative lessons; and methodological quality and reporting were extracted. Where possible, individual data was extracted. We excluded cases of delayed post-infectious sequelae that did not occur in the context of acute severe disease.

For the primary and grade-specific meta-analyses, outcome was defined as cumulative incidence of death or survival with neurological sequelae at maximum follow-up. MRI outcome subgroups included positive and negative image results in the first two weeks after disease onset. Outcomes for cognitive and developmental sequelae were calculated. We used a Freeman-Tukey Double Arcsine Transformation for stabilizing the variances before performing the pooled estimate. Assuming binomial distribution allowed inclusion of studies with proportions equal to zero.^23^ A random effects model was performed applying the DerSimonian and Laird method to deal with extra between-study variation.^24^ Heterogeneity was evaluated using the I-squared measure and the Cochrane test for heterogeneity (Q statistic). The I-squared was interpreted as high heterogeneity (>=75%), moderate heterogeneity (>=50%) and low heterogeneity (>=25%).^25^ A deeper exploration using collecting period and methodological and reporting quality was performed. Bias was evaluated with funnel plots. A protocol for this review was submitted to Prospero.^26^

The three funders of the study had no role in study design, data collection, data analysis, data interpretation, or writing of the report. The corresponding author had full access to all the data in the study and had final responsibility for the decision to submit for publication.

## 3 Results

Our search identified 2472 articles. Of these, 25 had adequate methodology and reporting for inclusion in quantitative analyses [Table 1], and 43 were included in the text review.

**Table 1:** List of studies included in the quantitative analysis.. For a list of studies included in text review, see Appendix 3.

First Author	Year of publication	Reference number	Study Country	Year(s) of data collection	Language	Study design	Definition of exposure	Mean/median (*) age (month)^§^	Male: Female ratio	Mean follow up (month)	Neurological Outcomes					MRI outcomes (n)	Cognitive outcomes	Development outcomes
											Primary	Grade IIa	Grade IIb/III (total)	AFP	Grade IV			
Chang	2007	15	Taiwan	1998–2003	English	Prospective	Culture/PCR	21.6	1.49	34.8	181	61	53	21			✓	✓
Li	2015	A	China	2008–2012	Chinese	Retrospective	Clinical	26.4	1.14	NR	126							
Zhang	2014	B	China	Jan 1 2010–Dec 31 2012	English	Prospective	Culture/PCR	NR	1.41	NR	89							
Shen	2014	53	China	2011–2013	Chinese	Retrospective	Clinical	NR	2.49	NR	75							
Li	2012	29	China	May 2008–Sept 2010	English	Prospective	Clinical	19*	1.33	NR	35	7	25	9	3			
Lou	2013	C	China	2011	Chinese	Retrospective	Culture/PCR	19	1.75	NR	33							
Liu	2012	D	China	2008–2010	Chinese	Retrospective	Culture/PCR	13*	1.69	NR	30	6	20	8	4			
Lee	2010	30	Taiwan	June 2006–June 2008	English	Retrospective	Culture/PCR	NR	NR	NR	21	1	14	3	6			
McMinn	2001	16	Australia	Feb–sept 1999	English	Retrospective	Culture/PCR	18.2 (18*)	0.44	3.4	9	4	5	2				
Tsou	2008	31	Taiwan	Oct 1997–June 2002	English	Retrospective	Culture/PCR	NR	NR	36			81		44		✓	✓
Liu	2015	32	China	2012–2013	Chinese	Retrospective	Clinical	25	2.15	NR			61		2			
Huang	2006	33	Taiwan	1998–2004	English	Prospective	Clinical	28.8	1.52	33.6			56		7		✓	✓
Zhang	2011	E	China	2008	Chinese	Retrospective	Clinical	16*	4	NR			20	20				
Chen	2013	34	China	May 2008–Sept 2010	English	Retrospective	Clinical	19*	1.33	NR			19		2			
Peng	2012	F	China	2011	Chinese	Prospective	Clinical	21.6	1.67	1			16	16				
Shen	1999	I	Taiwan	April–October 1998	English	Retrospective	Clinical	25	NR	3			15					
Lee	2014	42	Taiwan	Jun 1998–July 2012	English	Prospective	Clinical	33.5 (27*)	1.6	29.9			13	13		27		
Tsai	2014	35	Taiwan	2001–2006	English	Retrospective	Clinical	22*	1.71	NR			32		14			
Zhang	2013	G	China	2011–2012	Chinese	Retrospective	Clinical	21	1	NR			8	8				
Hsia	2005	36	Taiwan	May 2000–Sept 2001	English	Retrospective	Serology	11*	1.08	NR					27			
Fu	2014	H	China	2010–2011	Chinese	Retrospective	Not reported	19.8	0.89	NR					17			
Lee	2012	37	Taiwan	Oct 2000–June 2008	English	Prospective	Culture/PCR	17 (18*)	2.33	85.4					10		✓	✓
Nolan	2003	38	Australia	Dec 2000–May 2001	English	Prospective	Clinical	21 (17.5*)	1	NR					6	6		
Chen	2014	43	China	May 2008–Oct 2011	English	Prospective	Clinical	16 (13*)	1.4	NR						12		
Chen	2001	41	Taiwan	April–Dec 1998	English	Prospective	Clinical	13.1 (16*)	1.33	NR						7		
TOTALS:											599	79	438	100	142	52		

Table ranked by analysis group, number of participants and year of study.

§ Mean to one decimal place where available.

NR – not reported.

**References of studies not referenced in the body of the text**

A – Li L, Liang G, Li Z, et al. Clinical features and neuropsychological development follow up on 126 patients with hand, foot and mouth disease and encephalitis. General Practice of Chinese Medicine. 2015;13(4):607–8.

B – Zhang Q, MacDonald NE, Smith JC, et al. Severe enterovirus type 71 nervous system infections in children in the Shanghai region of China: clinical manifestations and implications for prevention. Paediatric Infectious Diseases Journal. 2014 May;33(5):482–7.

C – Lou Y, Jin H, Liang P. Follow-up analysis of severe hand, foot and mouth disease in 33 cases caused by enterovirus 71. Chinese Medical Science. 2013;13:211–2.

D – Liu K, Ma X, Zhang C, et al. MRI and clinical characteristics of follow-up study in patients with neurological complications of enterovirus 71-related hand, foot, and mouth disease. Chinese Journal of Medicine. 2012;92(25):1742–6.

E – Zhang L, Wang Y, Wang X, et al. Two-year clinical follow-up study in patients with acute flaccid paralysis of hand, foot and mouth disease. General Practice of China. 2011;14(20):2260–3.

F – Peng B, Du Z, Li X, et al. Evolution and prognosis of acute flaccid paralysis from MRI image in patients with enterovirus 71 infection. Chinese Journal of Paediatrics. 2012;50(4):255–60.

G – Zhang S, Jia L, Li G. Clinical analysis and follow-up on eight children with hand, foot and mouth disease and acute flaccid paralysis. Modern Practical Medicine. 2013;25(7):806–8.

H – Fu S, Luo X, Wen X, et al. Clinical analysis and follow-up study in children with severe hand, foot and mouth disease. General Practice of China. 2014;17(4):403–7.

I – W.C. Shen, H.H. Chiu, K.C. Chow and C.H. Tsai, MR imaging findings of enteroviral encephaloymelitis: an outbreak in Taiwan, AJNR Am J Neuroradiol 20, 1999, 1889–1895.

Nine studies with a total of 599 children, 59.8% male, were included in the primary analysis.

Estimated cumulative incidence of death or long-term neurological sequelae at maximum follow-up was 19.8% (95% CI:10.2%, 31.3%), Iˆ2 88.57%. First year of data collection (1998–2008 vs. 2010–2011) (p = 0.002) and reporting quality (p = 0.00) accounted for heterogeneity; methodological quality did not (p = 0.20). Only one study^15^ reported loss to follow-up, and accounting for this reduced Iˆ2–84.47% [Appendix 4]. Smaller studies were biased towards reporting higher incidence of poor outcomes, with one outlier^15^ [Appendix 4].

In our primary analysis, 8 studies described neurological outcomes (n = 524). Of these, 60 children died, 54 developed limb weakness, 19 ventilator dependence, 17 dysphagia, 10 ataxia, 9 facial nerve palsy, 4 seizures, 1 internuclear ophthalmoplegia, and 1 left arm and bilateral toe amputation after ECMO.

Risk of sequelae or death was 0.0% (0.0%, 0.0%) for grade IIa; 17.0% (7.9%, 28.2%) for grade IIb/III; and 81.6% (65.1%, 94.5%) for grade IV. Significant heterogeneity existed between these groups (p = 0.00), demonstrating an association between acute severity and sequelae or death.

There was low heterogeneity within grade IIa (Iˆ2 = 0.00%), high heterogeneity within grade IIb/III (Iˆ2 = 84.15%), and moderate heterogeneity within grade IV (Iˆ2 = 65.77%). Within grade IIb/III, reporting quality accounted for heterogeneity (p = 0.021); first year of data collection (p = 0.262) and methodological quality (p = 0.309) did not. Smaller studies were biased towards reporting higher incidence of poor outcomes in grade IIb/III, but reduced incidence of poor outcomes in grade IV [Appendix 4].

Of 79 children from 5 studies with grade IIa disease in the analysis, there were no instances of sequelae.

Of 15 studies including 438 children with grade IIb/III disease, 9 studies delineated outcomes by specific presentation (AFP, brainstem encephalitis, encephalomyelitis), and 6 provided grouped data. Of 100 children from 9 studies with grade IIb/III disease and AFP as part of their presentation, 1.0% died, 45.0% had residual weakness at maximum follow-up, and 54.0% fully recovered. One Taiwanese patient had weakness persisting 20 years after acute disease, exempting them from military service.^27^

Serial data demonstrating the resolution of limb weakness over time were rare and non-uniform, precluding quantitative analysis. One study with 24 children found that 71% recovered full power by 2–3 months, and 13% had residual weakness at 6 months. Recovery was distal to proximal, and reflexes and tone recovered in parallel with power.^28^

Children suffering from grade IV disease were more likely to suffer from negative outcomes than those with grade IIa-III disease [Table 2]. The use of ventilatory support in the context of neurogenic pulmonary oedema or pulmonary haemorrhage is commonly described.^29^, ^30^, ^31^, ^32^, ^33^, ^34^, ^35^, ^36^, ^37^, ^38^ One study describes 72 children with grade IIb-IV disease requiring endotracheal intubation. Whilst 58 of these children were extubated successfully, 14 required tracheostomy (mean intubation 7.5 and 28.6 days respectively). Five children suffered laryngotracheal injury and could not be decannulated despite regaining independent ventilatory function.^31^

**Table 2:** Cumulative incidence of death, neurological sequelae or sequelae-free survival at maximum follow-up, divided by maximum severity of acute disease.

	Number of studies	Pooled *n*	Cumulative incidence by outcome (95% confidence interval)		
			Death	Neurological sequelae at maximum follow-up	Free of neurological sequelae
Primary analysis	9	599	0.070 (0.035–0.114)	0.098 (0.033–0.186)	0.813 (0.719–0.894)
Grade IIa	5	79	0.000 (0.000–0.000)	0.000 (0.000–0.000)	1.000 (1.000–1.000)
Grade IIb/III	15	438	0.000 (0.000–0.005)	0.161 (0.069–0.275)	0.830 (0.718–0.921)
Grade IV	12	142	0.327 (0.197–0.468)	0.385 (0.230–0.550)	0.184 (0.055–0.349)

*cumulative incidence does not add to 1 due to rounding of data required by the statistical analysis.

In those surviving grade IV disease, duration and degree of hypotension, and high inotrope requirements, were associated with ventilator dependence.^36^ Younger age was associated with increased risk of ventilator requirement and, of those intubated, older age was associated with ventilator dependence.^31^^,^^36^.

One study describes 3 children with respiratory failure. One responded to phrenic nerve stimulation but two did not. The clinical distinction between respiratory centre damage and phrenic nerve dysfunction could be useful in guiding future treatment.^38^

Bulbar dysfunction and dysphagia are also commonly described. In one study, 10 children required gastrostomy, with six dying of poor nutrition and sepsis after an average of 3.6 months in intensive care.^31^ Pulmonary oedema was an independent risk factor for gastrostomy. Long-term NG tube feeding complicated by recurrent aspiration pneumonia is also described.^31^^,^^37^.

Other outcomes described in grade IV disease includes seizures, sometimes requiring long-term anticonvulsant therapy, and children with severe motor sequelae rendering them bed-bound.^37^

Four studies explicitly assessed cognitive and developmental outcomes [Appendix 5] using variable assessment tools and definitions of significant impairment.^15^^,^^31^^,^^33^^,^^37^ One paper found acute disease severity to be associated with cumulative incidence of poor developmental outcomes (p = 0.01); acute disease severity, age at onset, maternal and paternal educational level were associated with IQ score (p = 0.00, p = 0.01, p = 0.04 and p = 0.02).^15^

Two case–control studies found an association between HFMD and Attention-Deficit/Hyperactivity Disorder (ADHD). One prospectively assessed 86 children with severe HFMD 4–5 years after acute disease using the Conners' Parent and Teacher Rating Scales, finding that 20% of cases had raised scores compared to 3% of controls. Age of onset, clinical severity, MRI findings and laboratory data collected during hospitalisation did not predict ADHD symptom score.^39^ The other compared ADHD cases with healthy controls, finding that ADHD was associated with previous infection with EV-A71 and more strongly with severe EV-A71 disease.^40^

Fifty-one children from 4 studies^41^^,^^42^^,^^38^^,^^43^ had MRI performed within 2 weeks of disease onset with paired outcome data [Appendix 6]. Two, 33 and 16 children had grade IIa, IIb/III and IV disease respectively. Cumulative incidence of sequelae or death at maximum follow-up was 0.01 (0.00–0.07) following a negative MRI and 0.63 (0.33–0.89) if positive. Moderate study heterogeneity was observed in the positive MRI group.

Type of lesion, anatomical site of lesion and association between MRI findings and clinical outcomes are summarised in appendix 6. No study statistically tested associations between MRI and outcome.

Three studies compared outcomes following severe HFMD secondary to EV-A71 infection and other viral causes [Appendix 7]. In those with EV-A71 infection, one study found increased rates of death (p = 0.02),^46^ and two studies found increased rates of sequelae (p = 0.02, p = 0.00)^51^^,^^52^ compared to non EV-A71 infection.

Of 25 studies included in the quantitative analysis, 3 were prospective and the remaining retrospective; 15 defined cases clinically rather than by laboratory methods alone; 16 used a WHO grading system or similar; and 8 had follow-up of more than one year. Adequate reporting was seen in 8 studies for the inclusion pathway; 23 for case definition; and 4 for follow-up methodology [Appendix 2].

## 4 Discussion

Quantitative and qualitative hospital-based data from 6 countries between 1980^27^ and 2013^28^^,^^32^^,^^53^ were incorporated. The quantitative analysis included 25 studies and 1090 children. The cumulative incidence of sequelae-free survival at maximum follow-up after severe HFMD was 80.2% (95% CI: 68.7%, 89.8%), comparable to outcomes following bacterial meningitis where full recovery is seen in 83.6% (developed countries) and 73.5% (developing countries) of children (n = 4920).^54^ The burden of sequelae is concentrated in children suffering more severe acute disease (p = 0.00), especially grade IV disease.

The brainstem is “hard-wired” for physiological functions such as consciousness, breathing and blood pressure control.^55^^,^^56^ It plays an important role in mediating responses to the environment, with communication to the cerebellum, thalami, basal ganglia, motor cortex as well as limbic, emotional and attentive systems, which influence cognition, memory and learning.^57^ Brainstem lesions therefore influence a wide range of processes and functions, and manifestations will evolve with child development, especially higher functions (e.g. cognition) that are more easily evaluated at school age and beyond.^58^

Poor neurological, developmental and cognitive recovery after HFMD may manifest due to direct neuronal damage by viral invasion in the brainstem or higher brain centres,^47^^,^^59^^,^^33^^,^^60^^,^^61^^,^^46^ hypoxic injury due to CPF,^62^^,^^33^ central hypoventilation^38^ or phrenic nerve dysfunction.^38^ Developmental and cognitive recovery may also be impaired by environmental factors such as poor access to rehabilitation or school absence.^15^^,^^45^.

Sequelae such as limb weakness, facial nerve palsy, and cerebellar signs were described across subgroups grade IIb-IV. In contrast ventilator dependence, bulbar dysfunction and the presence of multiple physical disabilities rendering the child fully dependent were found almost exclusively in those with grade IV disease. Our results suggest that a negative MRI in the acute setting may be a good prognostic sign in children with grade IIb-III disease, though this is an uncommon finding. Use of MRI in grade IV disease is more questionable given the consistency of such findings and the challenges of performing a scan on clinically unstable children. No RCTs featured in the quantitative analysis since outcomes in survivors were not described.^63^,^64^ EV-A71 vaccine trials have provided evidence for their efficacy in reducing the risk of developing severe disease in the acute phase, but none yet for reducing death or sequelae.^63^

In Singapore, Chinese and Malay children are more susceptible to developing HFMD than Indian children, and HLA-A33 and HLA-A2 are associated with susceptibility to EV-A71 infection and progression to CPF respectively (p = 0.00, p = 0.03).^66^^,^^67^ It would follow that similar factors exist for sequelae following infection.

Heterogeneity between studies in the meta-analysis can be explained by population and viral factors creating unique outbreaks; variable thresholds for hospital admission; differences in standard of care (e.g. inotrope use); and differences in methodological and reporting quality of studies included, particularly regarding follow-up. For example, Chang and colleagues (2007)^15^ showed a higher proportion of sequelae or death than other studies and was the only prospective study excluding participants lost to follow-up in the primary analysis, individuals likely to have better outcomes. When the dataset was adjusted for this, heterogeneity reduced [Appendix 4].

Study methodology and reporting were variable, partly because long-term outcomes were often secondary outcomes in retrospective studies. Accurate descriptive data (age, gender) and time and duration of follow-up were often lacking, precluding analysis of these as risk factors. Some studies used diagnosis by laboratory identification of EV-A71 rather than clinical assessment [Appendix 2]. Most studies did not provide adequate clinical detail for grade IIb and III children to be distinguished, meaning data from these grades were merged.

Our analysis likely underestimated morbidity because children without overt sequelae at discharge were often not followed up and many studies only assessed direct neurological sequelae, failing to capture multi-domain developmental, cognitive or psychiatric impairments. Conversely, inadequate follow-up may have failed to capture children making delayed but full recoveries. Studies of grade IV disease from Australia were over-represented, likely because of the clinical and academic resources available.

Our study rigorously adhered to the PRISMA checklist and used robust statistical techniques for combining proportion data. It was innovative in approaching a bilingual dataset. Finally, this study is based on observational data from clinical setting making it valuable for prognostication. This is helped by the outcome measures of survival with sequelae or death that a clinician can use at the point of discharge.

There were some limitations to this study. Chinese studies were not included in the text review and no dedicated grey literature search was performed in Chinese. The time between literature search and publication was significant due to the bilingual and multinational methodology.

## 5 Conclusion

This systematic review and meta analysis demonstrates a substantial burden of long-term sequelae and death following acute severe HFMD associated with EV-A71 in East Asia. The authors propose a research agenda in order to discover the true burden of this neurotropic disease, including an urgent call for studies specifically designed to prospectively follow-up survivors with regular, validated assessment.

**Panel 1: research & policy recommendations**

• *Well-designed and reported prospective studies of the morbidity burden of severe HFMD.* Basic patient demographics should be collected and assessed in relation to outcomes. Patients should be assessed at discharge, and follow-up should continue serially until at least school age. Neurological, developmental, cognitive and psychiatric outcomes should be assessed using standardised tools with comparative control groups
• *RCTs of vaccinations and therapies for severe disease* should adhere to the principles outlined above including long-term side effects of interventions e.g. ECMO
• *Patients with grade IIb-III disease are potentially amenable to intervention to limit progression.* Grade III requires better identification with non-invasive monitoring. MRI in the acute setting may be of prognostic value. Assessment of seizures in this group are limited and subclinical seizures may impact prognosis, evaluation using electroencephalography (EEG) possibly required.
• *Associations between immunopathological syndromes, HFMD and long-term sequelae are weak and require exploration*
• *Pathophysiology including aetiological agents, mechanism of neuronal invasion causing CPF, secondary autoimmune effects requires further study* and would allow the assessment of candidate preventative, neuroprotective and supportive therapies. Animal models are needed.
• *Genome-wide association studies and prospective observational studies publishing individual data* could provide valuable information of the interplay between host factors (e.g. genetic variants associated with poor outcomes, age, and gender) and disease phenotype.
• *Cost-benefit analysis (including QALY/DALY calculations) of long-term interventions in these patients* could facilitate the distribution of health resources.

## Funding

HRvD and SS were funded by the Wellcome Trust of Great Britain (089276/Z/09/Z and 106680/Z/14/Z) and a Li Ka Shing Foundation–University of Oxford Global Health Program strategic award (LG17). JCBA has been awarded a doctoral scholarship by CIENCIACTIVA, an initiative of the Peruvian National Council of Science, Technology and Technological Innovation (CONCYTEC); grant contract number 231-2015-FONDECYT. HY was supported by the National Science Fund for Distinguished Young Scholars (No.81525023), the National Natural Science Foundation of China (No. 81473031), the Li Ka Shing Oxford Global Health Programme (No.B9RST00-B900.57).

## Statement of authorship

TP and EJ were involved in methodology, investigation, formal analysis, protocol and paper writing; FL, LL, CY, SZ, QC, YL, QL and HY were involved in methodology, investigation and editing final draft. JCBA was involved in methodology, statistical analysis and editing final draft. HRvD was involved in methodology, supervision, and editing final draft. SS was involved in conceptualisation, methodology, supervision, protocol and editing final draft.

## Conflict of interests

All authors report no conflicts of interests in the writing of this publication.
