# Supplementary material for: Outcomes following severe hand foot and mouth disease: A systematic review and meta-analysis
Source: Eur J Paediatr Neurol. 2018 Sep;22(5):763–73. doi: 10.1016/j.ejpn.2018.04.007 (PMC6148319; doi:10.1016/j.ejpn.2018.04.007)
Supplement: Multimedia component 1 [file mmc1.doc]

**Appendix 1 – search term**

|  | Population | Exposure | Outcome 1 | Outcome 2 |
| --- | --- | --- | --- | --- |
| **Medical Subject Headings**  **(MeSH)** | Paediatrics [MH] | Hand, Foot and Mouth Disease [MH] OR  Herpangina [MH] | Outcome assessment (health care) [MH] OR  Patient outcome assessment [MH] OR  Treatment outcome [MH] OR  Quality-Adjusted Life years [MH] OR  Prognosis [MH] OR  Follow-up studies |  |
| **Text words** | ***Validated hedge term for children:*** Infan* OR newborn* OR new-born* OR perinat* OR neonat* OR baby OR baby* OR babies OR toddler* OR minors OR minors* OR boy OR boys OR boyfriend OR boyhood OR girl* OR kid OR kids OR child OR child* OR children* OR schoolchild* OR schoolchild OR school child[tiab] OR school child*[tiab] OR adolescen* OR juvenil* OR youth* OR teen* OR under*age* OR pubescen* OR pediatrics[mh] OR pediatric* OR paediatric* OR paediatric* OR school[tiab] OR premature* OR preterm  http://www. ncbi.nlm.nih.gov/ pubmed/ 23084708 | OR HFMD OR "Hand foot and mouth disease" OR "Hand-foot-mouth disease" OR "Hand, foot, mouth disease" OR "Hand foot mouth disease" OR "Hand, foot mouth disease" OR "Hand, foot, and mouth" OR "Hand, foot, and mouth disease" OR EV71 OR HEV71 OR "EV 71" OR "enterovirus 71" OR EVA71 OR "EV A71" OR EV-A71 OR "Enterovirus A71" OR EV71 OR HEV71 OR "EV 71" OR "enterovirus 71" OR EVA71 OR "EV A71" OR EV-A71 OR "Enterovirus A71” OR CA16 OR "CA 16" CA-16 OR CVA16 OR "CVA 16" OR CVA-16 OR CV-A16 OR "coxsackie A16" OR "coxsackie 16" OR "coxsackie virus A16" OR "coxsackievirus A16" OR "coxsackie virus 16" OR "coxsackie A virus 16" OR CA6 OR "CA 6" CA-6 OR CVA6 OR "CVA 6" OR CVA-6 OR CV-A6 OR "coxsackie A6" OR "coxsackie 6" OR "coxsackie virus A6" OR "coxsackievirus A6" OR "coxsackie virus 6" OR "coxsackie A virus 6" OR CA10 OR "CA 10" CA-10 OR CVA10 OR "CVA 10" OR CVA-10 OR CV-A10 OR "coxsackie A10" OR "coxsackie 10" OR "coxsackie virus A10" OR "coxsackievirus A10" OR "coxsackie virus 10" OR "coxsackie A virus 10" | OR Outcome* OR follow-up OR follow up OR followed up OR following up OR sequel* OR prognos* OR longitudinal OR longterm OR long-term OR "long term" | Brain OR CNS OR central nervous system OR nervous system OR neurodevelopment* OR neuropsych* OR neurology* OR development* OR complication* OR behavio* OR cogniti* OR education OR litera* OR numer* OR motor OR focal OR weakness OR paralysis OR dysphagia OR *ventilation OR dysarthri* OR “cranial nerve” OR palsy OR ataxi* OR learning OR hearing OR vision OR visual OR eye-sight OR "eye sight" OR seizure* OR rehab OR rehabilitat* OR mental OR "mental health" OR psychiatr* OR psycholog* OR ADHD  Neuro-developmental assessment tools **below** will be included |

***Search term for neurodevelopmental assessment tools***

To broaden the search, names of standardised neurodevelopmental assessment tools have been used based on (…), with the search terms below.

“Mullen Scales of Early Learning” OR “Rapid Neurodevelopmental Assessment” OR “Reynell Developmental Language Scale” OR “Battelle Developmental Inventory” OR “Bayley Scales of Infant Development” OR “Griffiths Mental Development Scales” OR “Kilifi Developmental Inventory” OR “Ages and Stages Questionnaire” OR ASQ OR “MacArthur-Bates Communicative Development Inventories” OR “Adapted Developmental Milestones Checklist” OR “Vineland Adaptive Behavior Scales” OR “Developmental Screening Inventory” OR “Denver Developmental Screening Test” OR “A not B task” OR “Cambridge Neuropsychological Test Automated Battery” OR CANTAB OR “Digit Span” OR “Verbal Fluency Test” OR BSID OR “Peabody Picture Vocabulary Test” OR “Wisconsin card sorting test” OR “Kaufman Assessment Battery for Children” OR “McCarthy Scales of Children's Abilities” OR “Stanford Binet” OR “Wechsler Preschool and Primary Scale of Intelligence” OR “Woodcock-Johnson Tests of Cognitive Abilities” OR “Stroop-like Day-Night Task” OR “Adapted Developmental Milestones Checklist” OR “Ages and Stages Questionnaire” OR ASQ OR “AGS Early Screening Profiles” OR “Peg-Tapping Task” OR “Luria's tapping test” OR “Junior South African Individual Scales” OR “Cognitive Development Assessment” OR “A not B task” OR “Visual Search Task” OR “Conner's Continuous Performance Test” OR “Digit Span” OR “Early Grade Reading Assessment” OR “Go/No-Go Association Task” OR “Verbal Fluency Test” OR “California Verbal Learning Test” OR “Raven's Progressive Matrices” OR “Test of Everyday Attention for Children” OR “Universal Nonverbal Intelligence Test” OR UNIT OR “Wechsler Intelligence Scale for Children” OR “Wide-Range Achievement Test” OR “Childhood Career Development Scale” OR “Piper Fatigue Scale” OR “School-Entry Group Screening Measure” OR “Early Screening Profiles” OR “Ten Questions Questionnaire” OR “Behavior Rating Inventory of Executive Function” OR “Rey Auditory Verbal Learning Test” OR “Peg-Tapping Task” OR “Aptitude tests for School Beginners” OR “Junior South African Individual Scales” OR “n-Back Task” OR “Senior South African Individual Scales” OR ESSI OR “Reading and Spelling Test” OR “General Scholastic Aptitude Test” OR “Visual Search Task” OR “Raven's Progressive Matrices” OR “Rey Auditory Verbal Learning Test” OR “Visual Search Task” OR “Rapid Neurodevelopmental Assessment” OR “WHO Motor Development Milestones” OR “Adapted Developmental Milestones Checklist” OR “Developmental Screening Inventory” OR “time in Locomotion” OR “Total Motor Activity” OR “14 Gross Motor Milestones” OR “Bruininks-Osteretsky Test” OR “Adapted Developmental Milestones Checklist” OR “Movement Assessment Battery for Children” OR “Early Screening Profiles”

List of sources

| **English search engines** MEDLINE (pubmed)  Embase  Web of science (including conference procedings)  Cochrane library  Global health library (limited to developing country regions) - http://www.global healthlibrary.net /php/index.php | **Chinese search engines** China Hospital Knowledge Database  WANFANG (in chinese) | **Grey Literature sources** Dissertation abstracts international  Proquest dissertations and theses  Government agencies – World health organisation (WHO) (main and regional offices) and UN  WHO library    ***Clinical trials search***  Clinicaltrials.gov  WHO ICTRP (http://apps.who. int/trialsearch/ )  Cochrane clinical trial register |
| --- | --- | --- |
