# Supplementary material for: Outcomes following severe hand foot and mouth disease: A systematic review and meta-analysis
Source: Eur J Paediatr Neurol. 2018 Sep;22(5):763–73. doi: 10.1016/j.ejpn.2018.04.007 (PMC6148319; doi:10.1016/j.ejpn.2018.04.007)
Supplement: Multimedia component 2 [file mmc2.docx]

|  | **Methodology Assessment** | | | | | | | | | | **Reporting Quality** | | | | | | |  |
| --- | --- | --- | --- | --- | --- | --- | --- | --- | --- | --- | --- | --- | --- | --- | --- | --- | --- | --- |
| **First Author** | **Year** | **Study ID** | **Study type** | **Def. of exposure** | **Disease severity** | **Ax tool** | **Ax method** | **Average f/u** | **lost to f/u** | **Total** | **Epidemic?** | **inclusion pathway** | **case definition** | **age** | **Outcomes** | **CoI** | **Total** | **Text review only?** |
| Chang | 2008 | 340 | 2 | 2 | 3 | 0 | 0 | 4 | 0 | 11 | 0 | 2 | 3 | 0 | 0 | 1 | 6 |  |
| Chang | 2004 | 450 | 3 | 2 | 3 | 0 | 0 | 0 | 0 | 8 | 0 | 1 | 3 | 0 | 1 | 1 | 6 |  |
| Chang | 2007 | 385 | 3 | 2 | 3 | 3 | 2 | 4 | 2 | 19 | 0 | 3 | 3 | 1 | 4 | 1 | 12 |  |
| Chang | 2007 | 387 | - |  |  |  |  |  |  |  |  |  |  |  |  |  |  |  |
| Chang | 2004 | 496 | 3 | 2 | 1 | 1 | 2 | 3 | 0 | 12 | 0 | 3 | 3 | 1 | 1 | 1 | 9 |  |
| Chang | 1999 | 602 | 3 | 2 | 3 | 1 | 0 | 0 | 0 | 9 | 1 | 3 | 1 | 1 | 0 | 1 | 7 |  |
| Chang | 1999 | 604 | 3 | 2 | 3 | 0 | **0** | 0 | 0 | 8 | 1 | 2 | 3 | 1 | 0 | 1 | 8 |  |
| Chang | 2012 | 235 |  |  |  |  |  |  |  |  |  |  |  |  |  |  |  |  |
| Chen | 2001 | 555 | 3 | 3 | 3 | 1 | 2 | 4 | 0 | 16 | 1 | 2 | 3 | 1 | 1 | 0 | 8 |  |
| Chen | 2013 | 1407 | 3 | 3 | 3 | 1 | 2 | 4 | 0 | 16 | 1 | 2 | 3 | 1 | 1 | 1 | 9 |  |
| Chen | 2014 | 1405 | 3 | 3 | 3 | 1 | 2 | 4 | 0 | 16 | 1 | 3 | 3 | 1 | 1 | 1 | 10 |  |
| Chen | 2010 | 297 | 3 | 2 | 3 | 0 | 0 | 0 | 0 | 8 | 1 | 1 | 3 | 1 | 0 | 0 | 6 |  |
| Chi | 2013 | 149 |  |  |  |  |  |  |  |  |  |  |  |  |  |  |  |  |
| Chou | 2015 | 14 |  |  |  |  |  |  |  |  |  |  |  |  |  |  |  |  |
| Fu | 2003 | 495 |  |  |  |  |  |  |  |  |  |  |  |  |  |  |  |  |
| Gau | 2008 | 359 | 2 | 1 | 3 | 4 | 3 | 0 | 2 | 15 | 0 | 3 | 3 | 1 | 1 | 1 | 9 |  |
| Hsia | 2005 | 447 | 3 | 1 | 3 | 0 | 0 | 1 | 0 | 8 | 0 | 1 | 3 | 1 | 0 | 1 | 6 |  |
| Huang | 1999 | 586 | 3 | 2 | 3 | 1 | 2 | 2 | 0 | 13 | 1 | 1 | 3 | 1 | 1 | 1 | 8 |  |
| Huang | 2006 | 1311 | 4 | 3 | 3 | 3 | 4 | 0 | 0 | 17 | 0 | 1 | 3 | 1 | 4 | 1 | 10 |  |
| Hu | 2015 | 18 | 3 | 1 | 3 | 3 | 0 | 1 | 0 | 11 | 1 | 1 | 3 | 1 | 1 | 1 | 8 |  |
| Jan | 2010 | 308 | 3 | 1 | 3 | 3 | 2 | 3 | 0 | 15 | 0 | 2 | 3 | 1 | 3 | 1 | 10 |  |
| Jan | 2013 | 1619 |  |  |  |  |  |  |  |  |  |  |  |  |  |  |  |  |
| Lee | 2014 | 103 | 3 | 3 | 3 | 1 | 2 | 2 | 2 | 16 | 1 | 2 | 1 | 1 | 1 | 1 | 7 |  |
| Lee | 2012 | 218 | 3 | 2 | 3 | 3 | 2 | 4 | 2 | 19 | 1 | 2 | 3 | 1 | 1 | 0 | 8 |  |
| Lee | 2010 | 296 | 3 | 2 | 3 | 1 | 2 | 0 | 0 | 11 | 1 | 3 | 3 | 1 | 1 | 1 | 11 |  |
| Liao | 2001 | 2031 | 3 | 3 | 3 | 0 | 0 | 0 | 0 | 9 | 1 | 3 | 1 | 1 | 1 | 0 | 8 |  |
| Li | 2012 | 236 | 3 | 3 | 3 | 1 | 2 | 2 | 0 | 14 | 1 | 2 | 3 | 1 | 1 | 1 | 9 |  |
| Lo | 2011 | 263 | 3 | 2 | 3 | 1 | 0 | 0 | 0 | 9 | 1 | 2 | 3 | 1 | 1 | 1 | 9 |  |
| Lu | 2004 | 1196 | 3 | 2 | 3 | 1 | 2 | 3 | 2 | 16 | 1 | 3 | 3 | 1 | 1 | 1 | 10 |  |
| McMinn | 2001 | 543 | 3 | 2 | 3 | 1 | 2 | 1 | 0 | 12 | 1 | 3 | 3 | 1 | 0 | 0 | 8 |  |
| Nolan | 2003 | 853 | 3 | 3 | 3 | 0 | 2 | 4 | 1 | 16 | 1 | 3 | 3 | 1 | 0 | 0 | 8 |  |
| Ooi | 2003 | 497 |  |  |  |  |  |  |  |  |  |  |  |  |  |  |  | Y |
| Phan | 2012 | 1138 |  |  |  |  |  |  |  |  |  |  |  |  |  |  |  | Y |
| Prager | 2003 | 478 | 3 | 3 | 3 | 0 | 2 | 2 | 2 | 15 | 1 | 3 | 3 | 1 | 0 | 0 | 8 |  |
| Shen | 1999 | 582 | 3 | 3 | 3 | 0 | 0 | 0 | 2 | 11 | 1 | 2 | 3 | 0 | 0 | 1 | 7 |  |
| Suzuki | 2010 | 315 |  |  |  |  |  |  |  |  |  |  |  |  |  |  |  | Y |
| Tsai | 2014 | 121 | 3 | 3 | 3 | 3 | 2 | 0 | 1 | 15 | 0 | 2 | 3 | 1 | 3 | 1 | 10 |  |
| Tsai | 2004 | 441 |  |  |  |  |  |  |  |  |  |  |  |  |  |  |  | Y |
| Tsou | 2008 | 1077 | 3 | 2 | 1 | 3 | 2 | 4 | 1 | 16 | 1 | 3 | 3 | 1 | 4 | 0 | 12 |  |
| Wang | 2006 | 414 | 3 | 2 | 3 | 1 | 2 | 0 | 2 | 13 | 0 | 1 | 3 | 1 | 0 | 0 | 5 |  |
| Yang | 2005 | 425 |  |  |  |  |  |  |  |  |  |  |  |  |  |  |  | Y |
| Yingxue | 2012 | 1004 | 1 | 0 | 1 | 0 | 0 | 1 | 0 | 3 | 0 | 1 | 1 | 0 | 0 | 0 | 2 |  |
| Zhang | 2014 | 82 | 4 | 2 | 1 | 0 | 0 | 1 | 2 | 10 | 1 | 3 | 3 | 1 | 0 | 1 | 9 |  |
| Fu | 2014 | 3017 | 3 | 0 | 1 | 2 | 2 | 4 | 1 | 13 | 1 | 1 | 3 | 1 | 3 | 1 | 10 |  |
| Li | 2015 | 3033 | 3 | 3 | 1 | 4 | 2 | 3 | 2 | 18 | 1 | 1 | 3 | 1 | 3 | 0 | 9 |  |
| Liu | 2012 | 3040 | 1 | 2 | 1 | 1 | 2 | 1 | 2 | 10 | 1 | 2 | 3 | 1 | 1 | 1 | 9 |  |
| Liu | 2015 | 3042 | 1 | 3 | 3 | 1 | 2 | 2 | 2 | 14 | 1 | 1 | 3 | 1 | 1 | 0 | 7 |  |
| Lou | 2013 | 3046 | 3 | 2 | 1 | 2 | 2 | 2 | 2 | 14 | 1 | 3 | 1 | 1 | 1 | 0 | 7 |  |
| Peng | 2012 | 3052 | 4 | 3 | 1 | 2 | 2 | 1 | 2 | 15 | 1 | 2 | 3 | 1 | 3 | 0 | 10 |  |
| Shen | 2014 | 3056 | 1 | 3 | 3 | 2 | 2 | 1 | 0 | 12 | 1 | 1 | 3 | 1 | 1 | 0 | 7 |  |
| Zhang | 2011 | 3083 | 1 | 3 | 1 | 3 | 2 | 4 | 2 | 16 | 0 | 1 | 3 | 1 | 3 | 1 | 9 |  |
| Zhang | 2013 | 3085 | 1 | 3 | 1 | 3 | 3 | 2 | 2 | 15 | 0 | 1 | 3 | 1 | 4 | 0 | 9 |  |

**Scoring system**

|  | **Methodology Assessment** | | | | | | |  | **Reporting Quality** | | | | | |
| --- | --- | --- | --- | --- | --- | --- | --- | --- | --- | --- | --- | --- | --- | --- |
| **Score** | **Study type** | **Definition of exposure** | **Disease severity** | **Assessment tool** | **Assessment method** | **Average f/u** | **Lost to f/u** |  | **Epidemic?** | **Inclusion Pathway** | **Case Definition** | **Age** | **Outcomes** | **Conflict of Interest** |
| **4** | Interventional study or longitudinal | - | - | Control group + internationally recognised/locally standardised | - | >1 year | - |  | - | - | - | - | assessor, method, follow up process | - |
| **3** | Retrospective | Clinical definition | WHO/ similar | Internationally recognised | observed & reported | 6-12 months | - |  | - | diagram/ clear sentence | clear | - | two of these | - |
| **2** | Case - control | Culture / PCR | - | Locally standardised | observed | 3-6 months | <20% |  | - | some reference to | - | - | - | - |
| **1** | Case series/report | Serology | Any other | Non-standardised | reported | <3 months | >20% |  | outbreak/ background | final number only | ambiguous | reported | one of these | Funding source stated |
| **0** | - | Not reported or ambiguous (NR) | NR | NR | NR | NR | NR |  | NR | - | NR | NR | NR | NR |
