# Supplementary material for: Outcomes following severe hand foot and mouth disease: A systematic review and meta-analysis
Source: Eur J Paediatr Neurol. 2018 Sep;22(5):763–73. doi: 10.1016/j.ejpn.2018.04.007 (PMC6148319; doi:10.1016/j.ejpn.2018.04.007)
Supplement: Multimedia component 3 [file mmc3.docx]

Appendix 3 - Studies for Text Review

| First Author | Year of publication | Reference in manuscript | Journal | Study site | | Year(s) of data collection | Language |
| --- | --- | --- | --- | --- | --- | --- | --- |
|  |  |  |  | Country | Hospital |  |  |
| Chang | 2008 | 67 |  | Taiwan | Chang Gung Children's hospital | May 2001 - April 2003 | English |
| Chang | 2004 | not referenced | Paediatric Infectious Disease Journal | Taiwan | Chang Gung Children's hospital | 1998-2002 | English |
| Chang | 2007 | 27 |  | Taiwan | National Taiwan University Hospital | 1980-81 | English |
| Chang | 2004 | 45 |  | Taiwan | Chang Gung Children's hospital | Feb 2001 - Aug 2002 | English |
| Chang | 1999 | not referenced | Lancet | Taiwan | Chang Gung Children's hospital | April-July 1998 | English |
| Chang | 1999 | 46 |  | Taiwan | Chang Gung Children's hospital | April-Dec 1998 | English |
| Chang | 2012 | not referenced | Journal of medical virology | Taiwan | Chang Gung Memorial hospital | 2004/2005 & 2008 outbreaks | English |
| Chen | 2010 | 51 |  | Taiwan | Chang Gung Children's Hospital | 2008 | English |
| Chi | 2013 | 64 |  | Vietnam | Children's Hospital no. 1, HCMC | June 2007 - May 2010 | English |
| Chou | 2015 | 40 |  | Taiwan | Longitudinal Health Insurance Database | 2006-2010 | English |
| Fu | 2003 | 62 |  | Taiwan | ICU @ Taichung Veterans General Hospital | Jan 98 - Jan 01 | English |
| Gau | 2008 | 39 |  | Taiwan | Chang Gung Children's hospital & National Taiwan University Hospital | 1998-2003 | English |
| Huang | 1999 | 47 |  | Taiwan | Chang Gung Children's hospital | April - Nov 1998 | English |
| Hu | 2015 | 28 |  | China | Chongquing University of Medical Sciences - affiliated Children's Hospital | Jan - Dec 2013 | English |
| Jan | 2010 | 65 |  | Taiwan | Taichung Veterans General Hospital | 2000-2008 | English |
| Jan | 2013 | not referenced | International Journal Infectious Diseases | Taiwan | Taichung Veterans General Hospital | April-July 2012 | English |
| Liao | 2001 | 50 |  | Taiwan | Cathay General Hospital, Tapei | Feb 1998 - Jan 1999 | English |
| Lo | 2011 | not referenced | Journal of microbiology, immunology and infection | Taiwan | Chang Gung Children's Hospital | Jan 2004 - Dec 2009 | English |
| Lu | 2004 | 52 |  | Taiwan | Chang Gung Children's Hospital | Jan 2000 - Sept 2001 | English |
| Ooi | 2003 | not referenced | Clinical infectious diseases | Malaysia | Sibu Hospital, Sarawak | 1997 | English |
| Phan | 2012 | not referenced | Journal of medical case reports | Vietnam | National Hospital of Paediatrics in Hanoi | 2011 | English |
| Suzuki | 2010 | not referenced | Paediatrics international | Japan | (Nationwide) | 2000-2002 | English |
| Tsai | 2004 | not referenced | Neuropaediatrics | Taiwan | National Taiwan University Hospital |  | English |
| Wang | 2006 | 49 |  | Taiwan | Cheng Kung University Hospital | 1998-2003 | English |
| Yang | 2005 | not referenced | Journal of microbiology, immunology and infection | Taiwan | National Taiwan University Hospital | 1994-2003 | English |
| Yingxue | 2012 | not referenced | International Journal Infectious Diseases | China | Tianjin Children's Hospital |  | English |
