# Supplementary material for: Outcomes following severe hand foot and mouth disease: A systematic review and meta-analysis
Source: Eur J Paediatr Neurol. 2018 Sep;22(5):763–73. doi: 10.1016/j.ejpn.2018.04.007 (PMC6148319; doi:10.1016/j.ejpn.2018.04.007)
Supplement: Multimedia component 4 [file mmc4.doc]

***Appendix 4 – Statistical Analysis***

**1. Primary**

**DEATH + SEQUELAE**

**(Chang with N=232 – loss to follow up patients included in analysis for purpose of demonstrating reducing heterogeneity compared to N=181 analysis)**

**DETAH + SEQUELAE (HETEROGENEITY EXPLORATION)**

**(Chang with N=232)**

By PERIOD (1)

By PERIOD (2)

By METHOD

By REPORTING

**DEATH ONLY**

**(Chang with N=232)**

**WITH SEQUELAE ONLY**

**(Chang with N=232)**

**WITHOUT SEQUELAE ONLY**

**(Chang with N=232)**

**DEATH + SEQUELAE**

**(Chang with N=181 – loss to follow up patients excluded from analysis)**

**DETAH + SEQUELAE (HETEROGENEITY EXPLORATION)**

**(Chang with N=181)**

By PERIOD (1)

By PERIOD (2)

By METHOD

By REPORTING

**DEATH ONLY**

**(Chang with N=181)**

**WITH SEQUELAE ONLY**

**(Chang with N=181)**

**WITHOUT SEQUELAE ONLY**

**(Chang with N=181)**

**2. Subgroup**

**DEATH + SEQUELAE**

**DETAH + SEQUELAE (HETEROGENEITY EXPLORATION inside group GRADE IIb/III)**

By COLLECT PERIOD

By METHOD

By REPORTING

**DETAH ONLY**

**WITH SEQUELAE ONLY**

**WITHOUT SEQUELAE ONLY**

**3. Developmental and 4. Cognitive outcomes**

**COGNITIVE**

**DEVELOPMENTAL**

**5. MRI**

**MRI OPTION 2 (outcome= less seq + sev seq + death)**

**AFP**

**7. For Methods section:**

For drawing forest plots we used a routine specifically designed for pooling proportions in meta-analysis ([1](#_ENREF_1)). Using this tool, we performed a Freeman-Tukey Double Arcsine Transformation for stabilizing the variances before performing the pooled estimate, as well as an exact method -based on Clopper-Pearson limits and a Beta-distribution - for calculating confidence intervals of every study. We performed a random effects model with a DerSimonian and Laird method, which is recommended to deal with extra between studies variation ([2](#_ENREF_2)). For evaluating heterogeneity we used both, the I-squared measure and the Cochrane test for heterogeneity (Q statistic). The I-squared was interpreted following the recommendations of ([3](#_ENREF_3)): >=75% as high heterogeneity, >=50% as moderate heterogeneity and >=25% as low heterogeneity. A deeper exploration of potential heterogeneity sources such as collecting period, methodological quality of study and reporting, was also done. For evaluating bias, we performed funnel plots using studies sample sizes and proportions ([4](#_ENREF_4)).

**References**

1. Nyaga VN, Arbyn M, Aerts M. Metaprop: a Stata command to perform meta-analysis of binomial data. Archives of Public Health. 2014;72(1):1.

2. Deeks JJ, Altman DG, Bradburn MJ. Statistical methods for examining heterogeneity and combining results from several studies in meta‐analysis. Systematic Reviews in Health Care: Meta-Analysis in Context, Second Edition. 2008:285-312.

3. Higgins JP, Thompson SG, Deeks JJ, Altman DG. Measuring inconsistency in meta-analyses. Bmj. 2003;327(7414):557-60.

4. Hunter JP, Saratzis A, Sutton AJ, Boucher RH, Sayers RD, Bown MJ. In meta-analyses of proportion studies, funnel plots were found to be an inaccurate method of assessing publication bias. Journal of clinical epidemiology. 2014;67(8):897-903.
