# Supplementary material for: Outcomes following severe hand foot and mouth disease: A systematic review and meta-analysis
Source: Eur J Paediatr Neurol. 2018 Sep;22(5):763–73. doi: 10.1016/j.ejpn.2018.04.007 (PMC6148319; doi:10.1016/j.ejpn.2018.04.007)
Supplement: Multimedia component 5 [file mmc5.docx]

***Appendix 5 – Cognitive and Developmental Outcomes***

Developmental outcomes:

|  | Number of studies | n | Estimated risk of developmental delay (confidence interval) |
| --- | --- | --- | --- |
| Grade IIa | 1 | 43 | 0.00 (0.00-0.08) |
| Grade IIb/III | 3 | 157 | 0.04 (0.01-0.09) |
| Grade IV | 4 | 57 | 0.58 (0.04-1.00) |

Four studies explicitly assessed developmental outcomes[(Chang, Huang, et al. 2007; Huang et al. 2006; Lee et al. 2012; Tsou et al. 2008)](https://paperpile.com/c/qCIOdM/D662+GvUG+Dl67+RQVo). Assessment tools and definitions of significant developmental impairment varied between these studies; three used standardised tools. One study [(Chang et al. 2007)](https://paperpile.com/c/qCIOdM/D662) described outcomes for each developmental domain: of 22 children with developmental delay in total (21 WHO IV; 1 WHO IIb/III), 20 had gross motor delay, 18 had language delay, 16 had fine motor delay, and 16 had personal-social delay.

Acute disease severity was significantly associated with cumulative incidence of poor developmental outcomes (p=0.01)[(Chang, Huang, et al. 2007)](https://paperpile.com/c/qCIOdM/D662). A significant degree of heterogeneity existed in the 4 studies describing outcomes from grade IV disease (I^2=91.47%, p=0.00).

Cognitive outcomes:

|  | Number of studies | n | Estimated risk of cognitive impairment (%) |
| --- | --- | --- | --- |
| Grade IIa | 1 | 38 | 0.13 (0.04-0.28) |
| Grade IIb/III | 3 | 176 | 0.08 (0.04-0.13) |
| Grade IV | 4 | 38 | 0.13 (0.00-0.50) |

Four studies explicitly assessed for cognitive outcomes and were included in this estimate[(Chang, Huang, et al. 2007; Huang et al. 2006; Lee et al. 2012; Tsou et al. 2008)](https://paperpile.com/c/qCIOdM/D662+GvUG+Dl67+RQVo). All studies used a standardised IQ test, but cut-offs for significant cognitive impairment varied between studies. In this pooled estimate, there was no significant association between acute severity and cumulative incidence of cognitive outcomes (p=0.52). One study (Chang 2007) found that clinical severity, age at disease onset, and parental educational level were significantly associated with IQ score.

Summary of assessment tools used and definitions of impairments:

|  | Developmental outcome tool | Definition of impairment | Cognitive outcome tool | Definition of impairment |
| --- | --- | --- | --- | --- |
| [Chang, Huang, et al. 2007](https://paperpile.com/c/qCIOdM/D662+GvUG+Dl67+RQVo) | DDST II (for patients =<6) | Failing two or more test items that 75 to 90% of children of their age could pass, or failing one or more test items that more than 90% of children younger than their age could pass. | WISC III (for children >= 4 years) | Full scale IQ <85 |
| [Huang et al. 2006](https://paperpile.com/c/qCIOdM/D662+GvUG+Dl67+RQVo) | M-ABC and B-BDT for visual motor integration | <=5th percentile for age | WPPSI-R (3-6 years), WISC (6-16 years) | Scores <2 SD from mean |
| [Lee et al. 2012](https://paperpile.com/c/qCIOdM/D662+GvUG+Dl67+RQVo) | Assessment of developmental milestones | N/A | WISC-R (for children >=5 years) | Full scale IQ <85 |
| [Tsou et al. 2008](https://paperpile.com/c/qCIOdM/D662+GvUG+Dl67+RQVo) | M-ABC, B-BDT for visual motor integration | Failure to meet developmental milestones from 4-6 years of age | WPPSI-R and WISC-III | IQ <95% CI |
