# Supplementary material for: Outcomes following severe hand foot and mouth disease: A systematic review and meta-analysis
Source: Eur J Paediatr Neurol. 2018 Sep;22(5):763–73. doi: 10.1016/j.ejpn.2018.04.007 (PMC6148319; doi:10.1016/j.ejpn.2018.04.007)
Supplement: Multimedia component 6 [file mmc6.docx]

***Appendix 6 – Magnetic Resonance Imaging (MRI) Data***

Fifty-one children from 4 studies[^41^](https://paperpile.com/c/qCIOdM/774K)^,^[^42^](https://paperpile.com/c/qCIOdM/9K5r)^,^[^38^](https://paperpile.com/c/qCIOdM/vpKc)^,^[^43^](https://paperpile.com/c/qCIOdM/LfHI) had MRI performed within 2 weeks of disease onset with paired outcome data [Appendix 5]. Two, 33 and 16 children had grade IIa, IIb/III and IV disease respectively. Cumulative incidence of sequelae or death at maximum follow up was 0.01 (0.00-0.07) following a negative MRI and 0.63 (0.33-0.89) if positive. Moderate study heterogeneity was observed in the positive MRI group. Of 6 children with no intra-parenchymal findings on MRI in the acute setting, all had grade IIa-III acute disease, 5 completely recovered and 1 had a foot drop and mild shoulder weakness. Of 45 with positive MRI, 29 had grade IIb-III of whom 14 had prolonged sequelae at maximum follow up; and 16 had grade IV disease, all of whom had sequelae or died at maximum follow up.

Lesion site from 9 studies included the midbrain, pons and medulla of the brainstem, largely posteriorly[^37^](https://paperpile.com/c/qCIOdM/Dl67)^,^[^15^](https://paperpile.com/c/qCIOdM/D662)^,^[^34^](https://paperpile.com/c/qCIOdM/kznj)^,^[^43^](https://paperpile.com/c/qCIOdM/LfHI)^,^[^44^](https://paperpile.com/c/qCIOdM/o0HE); cerebellum;[^34^](https://paperpile.com/c/qCIOdM/kznj)^,^[^28^](https://paperpile.com/c/qCIOdM/q3lT)^,^[^16^](https://paperpile.com/c/qCIOdM/Aszg) and cervical to lumbar cord[^15^](https://paperpile.com/c/qCIOdM/D662)^,^[^41^](https://paperpile.com/c/qCIOdM/774K) including anterior horns and nerve roots including the sacral roots.[^41^](https://paperpile.com/c/qCIOdM/774K)^,^[^43^](https://paperpile.com/c/qCIOdM/LfHI)^,^[^34^](https://paperpile.com/c/qCIOdM/kznj) Widened subarachnoid space[^43^](https://paperpile.com/c/qCIOdM/LfHI) and meningeal enhancement[^29^](https://paperpile.com/c/qCIOdM/tYl4) were seen in association with aseptic meningitis.

Of 16 studies reporting MRI technique, hyperintensities in T2-weighted images were the most common positive finding.[^41^](https://paperpile.com/c/qCIOdM/774K)^,^[^15,16,31,34,35,38,45–49^](https://paperpile.com/c/qCIOdM/vpKc+D662+iyEq+Nhrf+kznj+GCPX+Aszg+b8hp+8Nj5+RQVo+wnNm)^,^[^43^](https://paperpile.com/c/qCIOdM/LfHI)^,^[^44^](https://paperpile.com/c/qCIOdM/o0HE). Hyper- and hypointensities in T1-weighted images,[^43^](https://paperpile.com/c/qCIOdM/LfHI)^,^[^29,34^](https://paperpile.com/c/qCIOdM/tYl4+kznj) diffusion-weighted images[^38^](https://paperpile.com/c/qCIOdM/vpKc)^,^[^50^](https://paperpile.com/c/qCIOdM/e53l) and enhancement in contrast scans[^41^](https://paperpile.com/c/qCIOdM/774K)^,^[^43^](https://paperpile.com/c/qCIOdM/LfHI)^,^[^34,47^](https://paperpile.com/c/qCIOdM/kznj+GCPX) were also identified.

Anatomical site of an MRI lesion is associated with the type of neurological sequelae.[^15^](https://paperpile.com/c/qCIOdM/D662)^,^[^43^](https://paperpile.com/c/qCIOdM/LfHI)^,^[^35^](https://paperpile.com/c/qCIOdM/8Nj5)^,^[^31^](https://paperpile.com/c/qCIOdM/RQVo) Extent of MRI change in the acute setting;[^47^](https://paperpile.com/c/qCIOdM/GCPX) and resolution versus persistence,[^41^](https://paperpile.com/c/qCIOdM/774K)^,^[^43^](https://paperpile.com/c/qCIOdM/LfHI)^,^[^44^](https://paperpile.com/c/qCIOdM/o0HE) atrophy or cavitation[^41^](https://paperpile.com/c/qCIOdM/774K)^,^[^47^](https://paperpile.com/c/qCIOdM/GCPX)^,^[^16^](https://paperpile.com/c/qCIOdM/Aszg)^,^[^44^](https://paperpile.com/c/qCIOdM/o0HE) of MRI lesions is associated with the degree of clinical improvement. In one study, “patch-like” hyperintensities carried a worse prognosis than “speckled” ones, n=21.[^34^](https://paperpile.com/c/qCIOdM/kznj) The ‘strength’ of MRI signal intensity;[^43^](https://paperpile.com/c/qCIOdM/LfHI) single versus multiple abnormalities in the brainstem and/or spinal cord;[^43^](https://paperpile.com/c/qCIOdM/LfHI) and the level of the spinal cord lesion are all associated with outcomes.[^43^](https://paperpile.com/c/qCIOdM/LfHI) No studies have statistically assessed these relationships.

| First Author | Year of publication | MRI in acute setting linked to outcomes? | MRI findings | complete recovery | less severe sequelae | severe sequelae / death | IIa | IIb-III | IV | T1 | T2 | Contrast |
| --- | --- | --- | --- | --- | --- | --- | --- | --- | --- | --- | --- | --- |
| Chen | 2001 | 7 | positive | 3 | 3 | 0 | 0 | 6 | 0 |  | 4 | 2 |
|  |  |  | negative | 1 | 0 | 0 | 0 | 1 | 0 |  |  |  |
| Lee | 2014 | 26 | positive | 6 | 13 | 4 | 0 | 14 | 9 |  |  |  |
|  |  |  | negative | 2 | 1 | 0 | 0 | 3 | 0 |  |  |  |
| Nolan | 2003 | 6 | positive | 0 | 2 | 4 | 0 | 0 | 6 | 0 | 6 |  |
|  |  |  | negative | 0 | 0 | 0 | 0 | 0 | 0 |  |  |  |
| Chen | 2014 | 12 | positive | 6 | 3 | 1 | 0 | 9 | 1 | 6 | 6 | 1 |
|  |  |  | negative | 2 | 0 | 0 | 2 | 0 | 0 |  |  |  |
| total | | 51 | positive | 15 | 21 | 9 | 0 | 29 | 16 |  |  |  |
|  |  |  | negative | 5 | 1 | 0 | 2 | 4 | 0 |  |  |  |

Lee 2014 - one patient didn't have MRI performed
