# Supplementary material for: Outcomes following severe hand foot and mouth disease: A systematic review and meta-analysis
Source: Eur J Paediatr Neurol. 2018 Sep;22(5):763–73. doi: 10.1016/j.ejpn.2018.04.007 (PMC6148319; doi:10.1016/j.ejpn.2018.04.007)
Supplement: Multimedia component 7 [file mmc7.docx]

***Appendix 7 – Data on Aetiological Agent***

Five studies looked at outcomes following infection with other viral agents. In each article, reference was made to a clinical spectrum that included HFMD.

| - | EV-A71 cases (WHO IIa+) | Death (%) | Sequelae (%) | Non EV-A71 cases (WHO IIa+) | Death (%) | Sequelae (%) | Comments |
| --- | --- | --- | --- | --- | --- | --- | --- |
| Chang 1999[^46^](https://paperpile.com/c/qCIOdM/Nhrf) | 57_a_ | 21 (36.8%) | 7 (17.5%) | 4 (CV-A16) | 0 (0.0%) | 0 (0.0%) | Significant difference in rates of death between groups (p=0.02) |
| Chen 2010[^50^](https://paperpile.com/c/qCIOdM/wC9r) | 18_b_ | 1 (5.6%) | 6 (33.3%) | 2 (CV-A2) | 0 (0.0%) | 0 (0.0%) | Significant difference in rates of sequelae between groups (p=0.02). |
| Lu 2004[^51^](https://paperpile.com/c/qCIOdM/ribM) | 46_a_ | 9 (19.6%) | 18 (39.1%) | 157_c_ | 2 (1.3%) | 0 (0.0%) | Significant difference in rates of sequelae (p=0.00) between groups. |
| Lo 2011[^52^](https://paperpile.com/c/qCIOdM/NTEc) | - | - | - | 3 (CV-A6) | 0 (0.0%) | 0 (0.0%) |  |
| Ooi 2003[^53^](https://paperpile.com/c/qCIOdM/JQJG) | - | - | - | 8 (Adenovirus 21) | 0 (0.0%) | 3 (37.5%) | All patients presented with AFP. Occurred during EV-A71 outbreak, and authors postulate that patients may have been co-infected. |

**Table 3 - summary of reports of severe HFMD associated with viruses other than EV-A71**

a,b: Certain EV-A71 cohorts are duplicates of data included in Chang 2007_(a)_ or Lee 2010_(b)._

c: includes 15 patients with grade IIb or more severe disease, of which 6 untypable enterovirus, 3 Cox A16, 1 Cox A9, and 5 enterovirus B infections (including 2 fatal cases in neonates).
