# Supplementary material for: Outcomes following severe hand foot and mouth disease: A systematic review and meta-analysis
Source: Eur J Paediatr Neurol. 2018 Sep;22(5):763–73. doi: 10.1016/j.ejpn.2018.04.007 (PMC6148319; doi:10.1016/j.ejpn.2018.04.007)
Supplement: Multimedia component 8 [file mmc8.docx]

***Index of supplementary data***

Appendix 1 – Systematic Review Search Term & List of Sources

Appendix 2 – Methodology Assessment and Reporting Quality of Included Studies

Appendix 3 – Studies for Text Review

Appendix 4 – Statistical Analysis

Appendix 5 – Cognitive and Developmental Outcomes

Appendix 6 – Magnetic Resonance Imaging Data

Appendix 7 – Data on Aetiological Agent
